# Intracranial migration of intraocular silicone oil mimicking metastatic disease

**DOI:** 10.1016/j.radcr.2020.12.058

**Published:** 2020-12-30

**Authors:** Nurahmed Mohammed, Daniel Urbine

**Affiliations:** Department of Medicine, Division of Pulmonary, Critical Care and Sleep Medicine, University of Florida, College of Medicine, FL, USA

**Keywords:** Intraocular silicone, Intracranial silicone, Retinal detachment, Intraventricular hemorrhage, Intracranial hemorrhage

## Abstract

Silicone oil (SO) is a commonly used agent of intraocular endotamponade for treating complicated retinal detachment. We report a case of SO migration into the cerebral ventricles which was initially misdiagnosed as metastatic disease. Misinterpretation of SO as metastatic disease in a patient with a lung nodule triggered admission to a medical intensive care unit and unnecessary evaluation with further imaging and invasive procedures.

## Case Presentation

A 58-year-old male presented with left-sided headache after inadvertently colliding with a door and hitting the left side of his face. The patient's past medical history was significant for mechanical mitral valve on anticoagulation, HLA-B27 associated uveitis, and bilateral retinal detachments. Four years ago, he underwent prosthetic replacement of the right eye and silicone oil (SO) endotamponade on the left, however this maneuver was ultimately unsuccessful, and he had no vision in either eye at baseline. Physical exam was notable for an irregular and non-reactive left pupil with no light perception; clean prosthetic within the right orbit; and a small abrasion on the left forehead and nose with minimal ecchymosis. His neurological exam was otherwise unremarkable.

The patient's initial CT head was misinterpreted as favoring intracranial metastasis which led to further work up with MRI head, CT chest, abdomen and pelvis. He was found to have a 13 mm spiculated nodule in the right lower lobe of the lung with low uptake on a positron emission tomography scan that also revealed other nonspecific findings in the gastrointestinal tract.

The patient was admitted to the intensive care unit where his anticoagulation, warfarin with therapeutic level at the time of admission, was held for concern of intracranial hemorrhage. He underwent an esophagogastroduodenoscopy and colonoscopy given the abnormal positron emission tomography scan findings.

He was seen by multiple services. While there were initial plans to obtain a surgical biopsy of his lung nodule, image review with radiology led to a prone CT head which confirmed the mobile debris in the ventricular system was in fact SO. Supine CT head revealed new (compared to a prior imaging two years earlier performed when he was presented to an outside emergency room with dizziness) hyperdense nodules in the anti-dependent portions of the lateral ventricles. MRI head with and without contrast showed the hyperdense lesions in the anti-dependent portions of the lateral ventricles demonstrated no appreciable enhancement but did demonstrate significant chemical shift artifact consistent with intraventricular migration of intraocular silicone. A prone CT head confirmed that the hyperdense intraventricular debris was mobile, supporting the suspicion of intraventricular migration of silicone.

While the patient was largely asymptomatic, misdiagnosis of intracranial SO migration triggered the unnecessary use of resources such as specialist consultation, intensive care admission, further imaging in addition to exposing the patient to potentially life-threatening complications by withholding anticoagulation. There was also a plan for lung resection, assuming the lung nodule represented a neoplastic process. After successful diagnosis, the patient's subsequent hospital course was uneventful.

## Discussion

Retinal detachment is a condition that affects approximately [1] in 10,000 people every year. Treatments for retinal detachment include SO endotamponade. Some complications associated with intraocular SO endotamponade include keratopathy, glaucoma, cataract, optic neuropathy, and subretinal migration of oil droplets [1,2]. An uncommonly reported complication has been the migration of SO along the optic nerve into the cerebral ventricular system. This was first documented by Williams et al in 1999 [3]. Since then, 22 cases of intraventricular SO have been reported in the literature. Because it appears hyperdense on CT images and hyperintense on T1-weighted MRI, [4], [5], [6] SO that has migrated to the ventricles can be diagnosed mistakenly as hemorrhage or neoplastic disease. This can lead to unnecessary use of resources, and invasive investigations. Because there is normally no direct communication between the vitreous humor and the CSF spaces of the brain and optic nerve, several explanations for this migration have been proposed: congenital anatomic variants [7], macrophage phagocytosis and migration [8], and increased intraocular pressure [9]. Most case reports detailing intraventricular migration of SO were incidental and without neurological complication. Some case reports have documented migrated SO found as a result of patients presenting with novel or worsening headaches. Reports of seizures also are present sporadically in the literature.

**Fig. 1:**
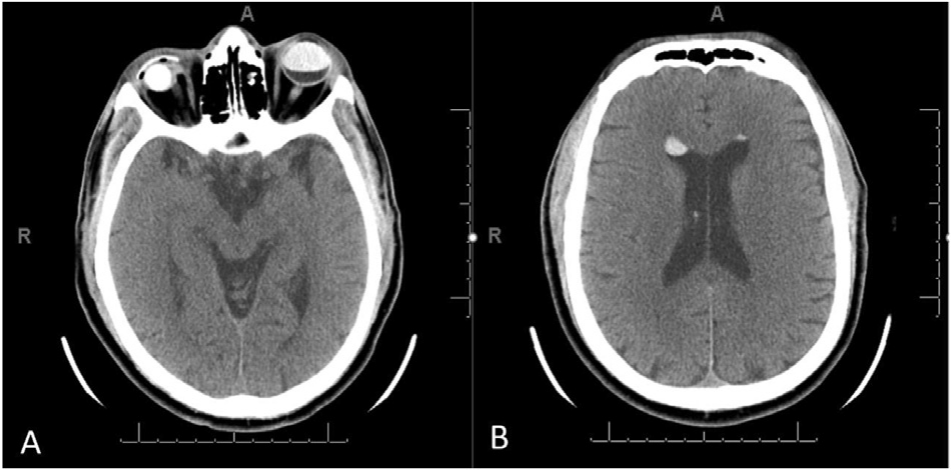
(A) Axial CT scan of the head at the level of the eyes showing changes from a right eye evisceration and acrylic conformer insertion in the past and an ovoid hyperdensity in the left globe that corresponds to intraocular silicone oil, which was used to endotamponade a previous retinal detachment. (B) Axial CT scan of the head at the level of the lateral ventricles showing 2 ovoid hyperdensities in the frontal horns bilaterally. The hyperdensities are located anteriorly, because the patient is supine, and the silicone oil has lower density than the cerebrospinal fluid.

**Fig. 2:**
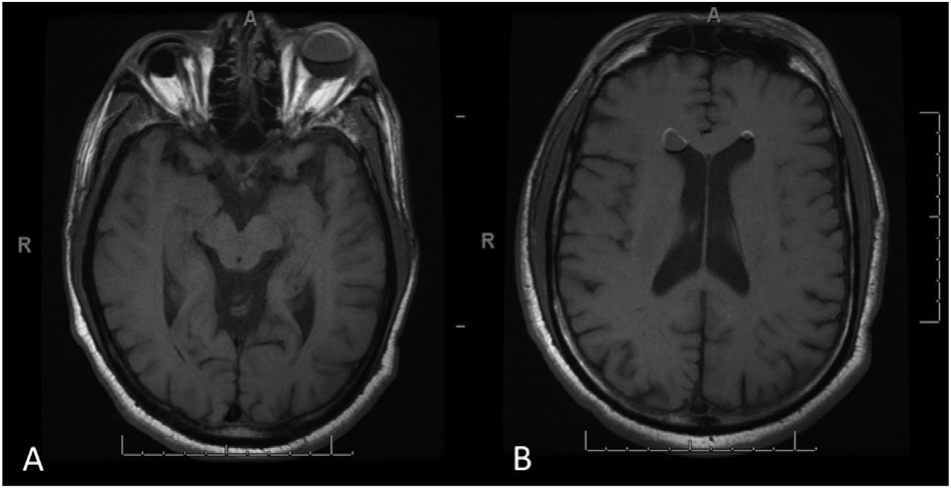
(A) Axial T1-weighted MRI at the level of the eyes showing again the previously performed right eye evisceration and acrylic conformer insertion, and an ovoid-shaped isointense signal in the left globe that corresponds to intraocular silicone oil and has the same signal signature with fat. (B) Axial T1-weighted MRI at the level of the lateral ventricles showing 2 ovoid hyperdensities in the frontal horns bilaterally.

**Fig. 3:**
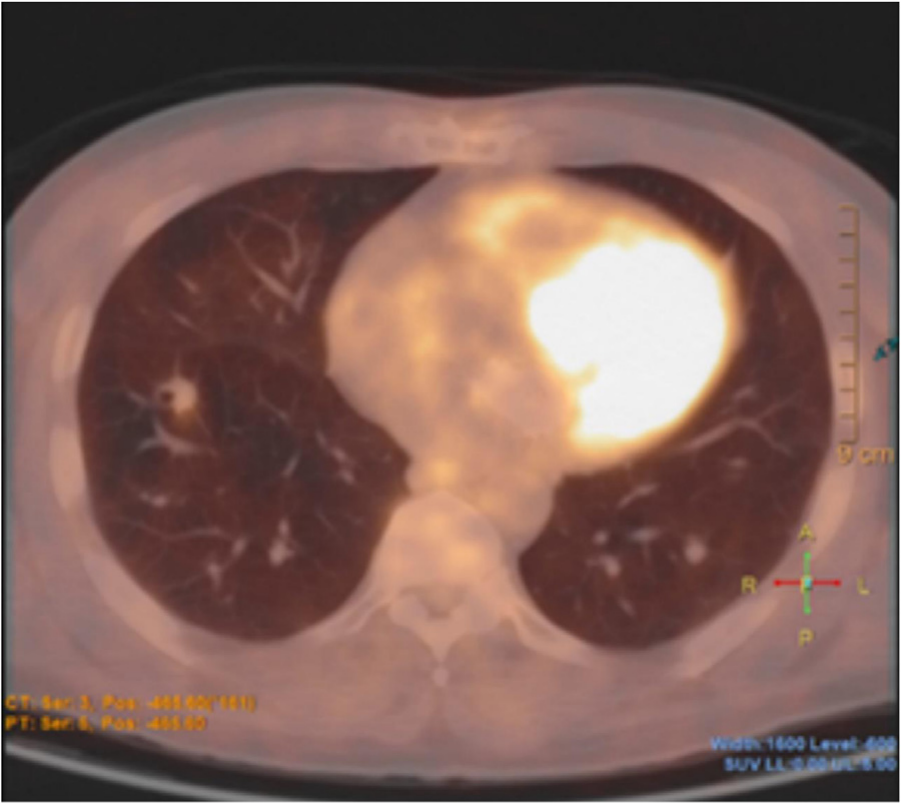
PET scan shows a 13 mm spiculated right lower lobe nodule with low FDG uptake.

**Fig. 4:**
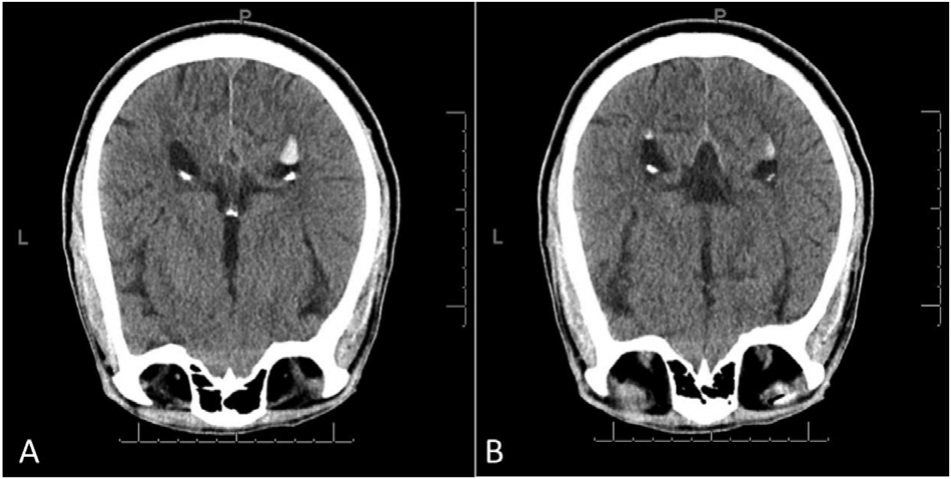
Prone head NCCT shows the hyperattenuating substance located in the occipital horns of the lateral ventricles moved from a gravity nondependent location to another gravity nondependent, which confirms the relative lower specific gravity of the hyperattenuating substance, consistent with silicone oil.

There are no specific published guidelines for the management of patients with intraventricular SO, necessitating a case-by-case approach for the care of these patients. In most cases, patients with intraventricular SO who presented with symptoms were successfully treated with conservative management. However, one case report describes a patient who presented with headache and third ventricular involvement, with increased intracranial pressure necessitating ventriculoperitoneal shunt catheter placement, with subsequent improvement of symptoms [10]. The presence of intracranial SO poses a potential risk for misdiagnosis on imaging with potential for further invasive investigation. This provides both an important complication for clinicians to recognize, as well as a reminder that cerebrospinal fluid spaces may have more extensive communication with other body compartments than often appreciated. Thorough imaging, meticulous history-taking, and assessment of patient presentation help ensure a correct diagnosis in the rare cases of SO migration to the cerebral ventricles. Because the appearance of SO can mimic hemorrhage or other pathologies on CT and MRI, careful comparison with prior imaging studies and prone non-contrast CT head are needed to avoid unnecessary follow-up studies (Fig. 1, Fig. 2, Fig. 3, Fig. 4)

## Conclusion

Our case adds to the limited body of knowledge surrounding SO migration into the central nervous system ventricular system. Clinicians should be aware of this complication, because of its potential confusion for hemorrhage and neoplastic disease. Thorough imaging, meticulous history-taking, and assessment of patient presentation help ensure a correct diagnosis in the rare cases of SO migration to the cerebral ventricles. Most patients are relatively asymptomatic on presentation. Others have presented with novel or worsening headaches and rarely seizure. Treatment depends on affected structures but typically is conservative.

## Consent

Patient gave a consent for this publication. There is also no patient identifying information in this publication.

## Declaration of Competing Interest

I have no conflict of interest.

## References

[1] Riedel KG, Gabel VP, Neubauer L, Kampik A, Lund OE. (1990). Intravitreal silicone oil injection: complications and treatment of 415 consecutive patients. Graefes Arch Clin Exp Ophthalmol Albrecht Von Graefes Arch Klin Exp Ophthalmol..

[2] Hruby PM, Poley PR, Terp PA, Thorell WE, Margalit E (2013). Headaches secondary to intraventricular silicone oil successfully managed with ventriculoperitoneal shunt. Retin Cases Brief Rep.

[3] Williams RL, Beatty RL, Kanal E, Weissman JL. (1999). MR imaging of intraventricular silicone: case report. Radiology.

[4] Herrick RC, Hayman LA, Maturi RK, DiazMarchan PJ, Tang RA, Lambert HM. (1998). Optimal imaging protocol after intraocular silicone oil tamponade. AJNR Am J Neuroradiol.

[5] Mathews VP, Elster AD, Barker PB, Buff BL, Haller JA, Greven CM. (1994). Intraocular silicone oil: in vitro and in vivo MR and CT characteristics. AJNR Am J Neuroradiol.

[6] Zhong H, Bianchi CM, Patel SJ, Wolfe AR, Visvikis GA. (2019). Intracranial migration of intraocular silicone oil following repetitive head trauma. Radiol Case Rep.

[7] Eller AW, Friberg TR, Mah F. (2000). Migration of silicone oil into the brain: a complication of intraocular silicone oil for retinal tamponade. Am J Ophthalmol.

[8] Papp A, Toth J, Kerenyi T, Jackel M, Suveges I. (2004). Silicone oil in the subarachnoid space—a possible route to the brain?. Pathol Res Pract.

[9] Shields CL, Eagle RC (1989). Pseudo-Schnabel's cavernous degeneration of the optic nerve secondary to intraocular silicone oil. Arch Ophthalmol.

[10] Federman JL, Schubert HD. (1988). Complications associated with the use of silicone oil in 150 eyes after retina-vitreous surgery. Ophthalmology.

